# Subclavian artery injury secondary to blunt trauma successfully managed by median sternotomy with supraclavicular extension: A case report and literature review

**DOI:** 10.1016/j.amsu.2020.03.012

**Published:** 2020-04-08

**Authors:** Adel Elkbuli, Kyle Kinslow, Brianna Dowd, Mark McKenney, Dessy Boneva, John Whitehead

**Affiliations:** a Department of Surgery, Kendall Regional Medical Center, Miami, FL, USA; b University of South Florida, Tampa, FL, USA; c Department of Surgery, Aventura Hospital and Medical Center, Miami, FL, USA

**Keywords:** Subclavian artery injury, Blunt trauma, Median sternotomy, Vascular repair, Trauma outcomes

## Abstract

**Introduction:**

Subclavian artery injury secondary to blunt trauma is rare and only a few cases have been documented in the literature. Subclavian arteries are protected by the clavicles, ribs, and chest wall. Clinical management and surgical approach vary depending on the specific injury. We present the case of a 50 year old male with blunt right subclavian transection.

**Case presentation:**

A 50-year-old male presented after being struck by a train. On exam, the patient had open injuries to the right upper chest/extremity. CTA showed a transection of the mid right subclavian artery along with a long traumatic occlusion distal to the defect. The patient was taken to the operating room where median sternotomy with supraclavicular extension was used to expose the transected ends of the subclavian artery and successfully perform a bypass graft. After a long hospital stay, he had a near-full functional recovery.

**Discussion:**

Blunt subclavian injury is rare and carries a high mortality. Adequate intervention requires prompt identification and proper surgical approach for repair. Median sternotomy offers the best approach to visualize the proximal right subclavian artery. Extension with a supraclavicular incision can be necessary for distal control. This approach offered timely intervention, which ultimately saved his life and allowed for return of pre-trauma functional status.

**Conclusion:**

Prompt identification of subclavian artery injury is paramount as such injuries carry a high mortality. Median sternotomy with supraclavicular extension is an appropriate open surgical approach to successfully manage proximal right subclavian artery injuries.

## Highlights

• Proximal right subclavian artery injuries secondary to blunt trauma are rare and often associated with significant comorbidities and higher mortality.
• Prompt surgical intervention adequately prevented death in our patient and allowed for full functional recovery.
• Management of such subclavian artery injuries typically require open surgical intervention given the extent of damage.

## 1 Introduction

**Fig. 1:**
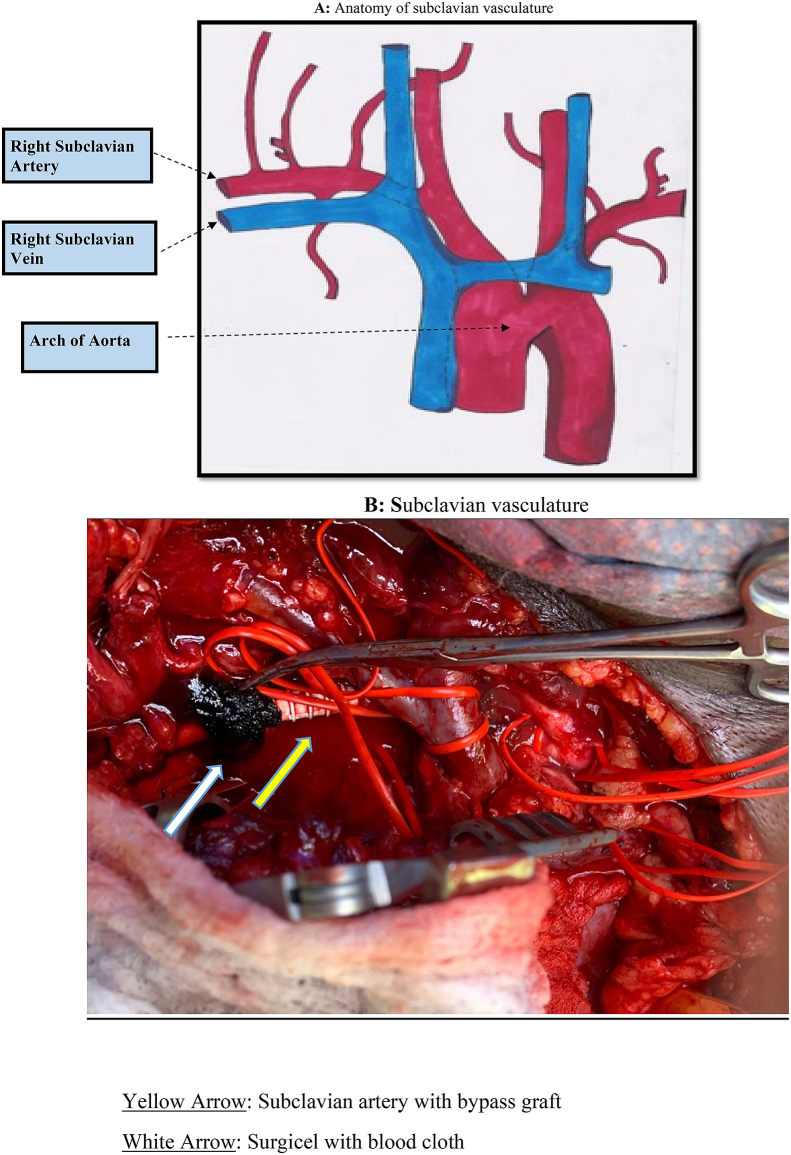
A: Anatomy of subclavian vasculature Figure 1BSubclavian vasculature.

Subclavian artery injury secondary to blunt trauma is a rare occurrence and only a few cases have been documented in the literature [[1], [2], [3], [4], [5]]. Much more commonly, subclavian artery injuries arise from penetrating mechanisms (ie. GSW or knife) or as a consequence of iatrogenic injury during central catheter placement [4]. The right subclavian artery is a continuation of the innominate artery, which branches directly from the aortic arch (Fig. 1). This artery has its own secondary distribution including the vertebral, internal thoracic, and thyrocervical trunk arteries, with its course continuing to form the brachial artery and its derivatives. The subclavian vessels are the main conduits responsible for blood supply to both upper extremities and when injured, can lead to severe hemorrhage and death by exsanguination. The proximal areas of both the right and left subclavian arteries are well surrounded by the clavicles, ribs, and chest wall leading to its great protection in the case of blunt trauma. This contrasts that of the more commonly seen distal subclavian/brachial injuries which are a consequence of their limited skeletal protection. In the rare occurrence of proximal subclavian injury secondary to blunt trauma, additional severe injuries such as rib fractures, brachial plexus injury, clavicular fractures, pneumothorax, pulmonary contusion, and traumatic brain injury are often present. Depending on the extent, mechanism, and position of the injury to the subclavian vessel, the technical management to stabilize the resulting hemorrhage varies (open repair vs. endovascular) as well as the open surgical approach (median sternotomy vs. thoracotomy). We present the case of a 50-year-old male who presented with a right subclavian transection secondary to blunt trauma after being struck by a train. This work has been reported in line with the SCARE criteria [6].

## 2 Case Presentation

**Fig. 2:**
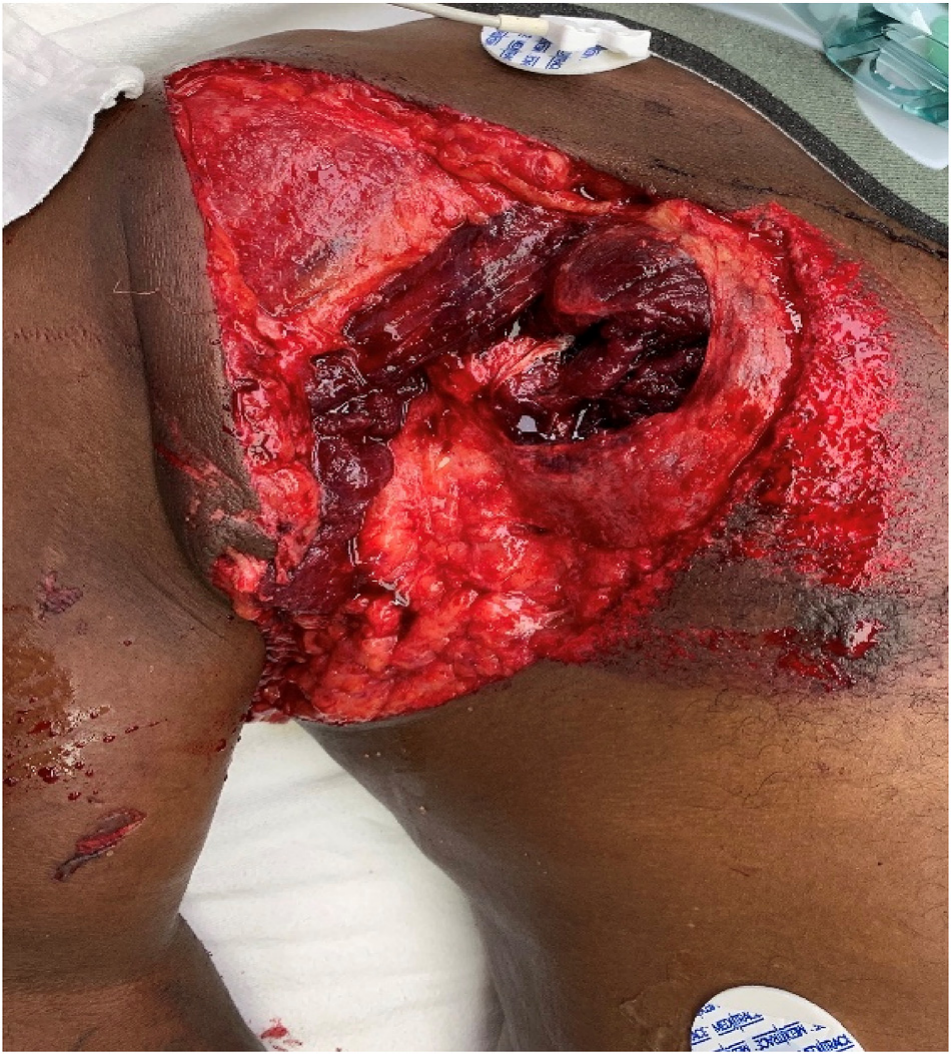
Large open right shoulder/thoracic soft tissue wounds exposing a transected pectoral major muscle.

A 50-year-old male patient presented to our Trauma Center after being struck by a train. Per Emergency Medical Services (EMS), he was attempting to cross the railroad tracks when he was struck. The patient had a significant past medical history of schizophrenia and did not recall the events leading to his accident but stated he was not suicidal. On initial examination, the patient had a GCS of 15 and noted to have a large open right shoulder/thoracic soft tissue wound exposing a transected pectoral major muscle along with palpable dislocation of the right shoulder (Fig. 2). Initial examination and confirmatory Doppler ultrasound exhibited an absent pulse signal in the right hand. He was unable to move his right arm entirely.

**Fig. 3:**
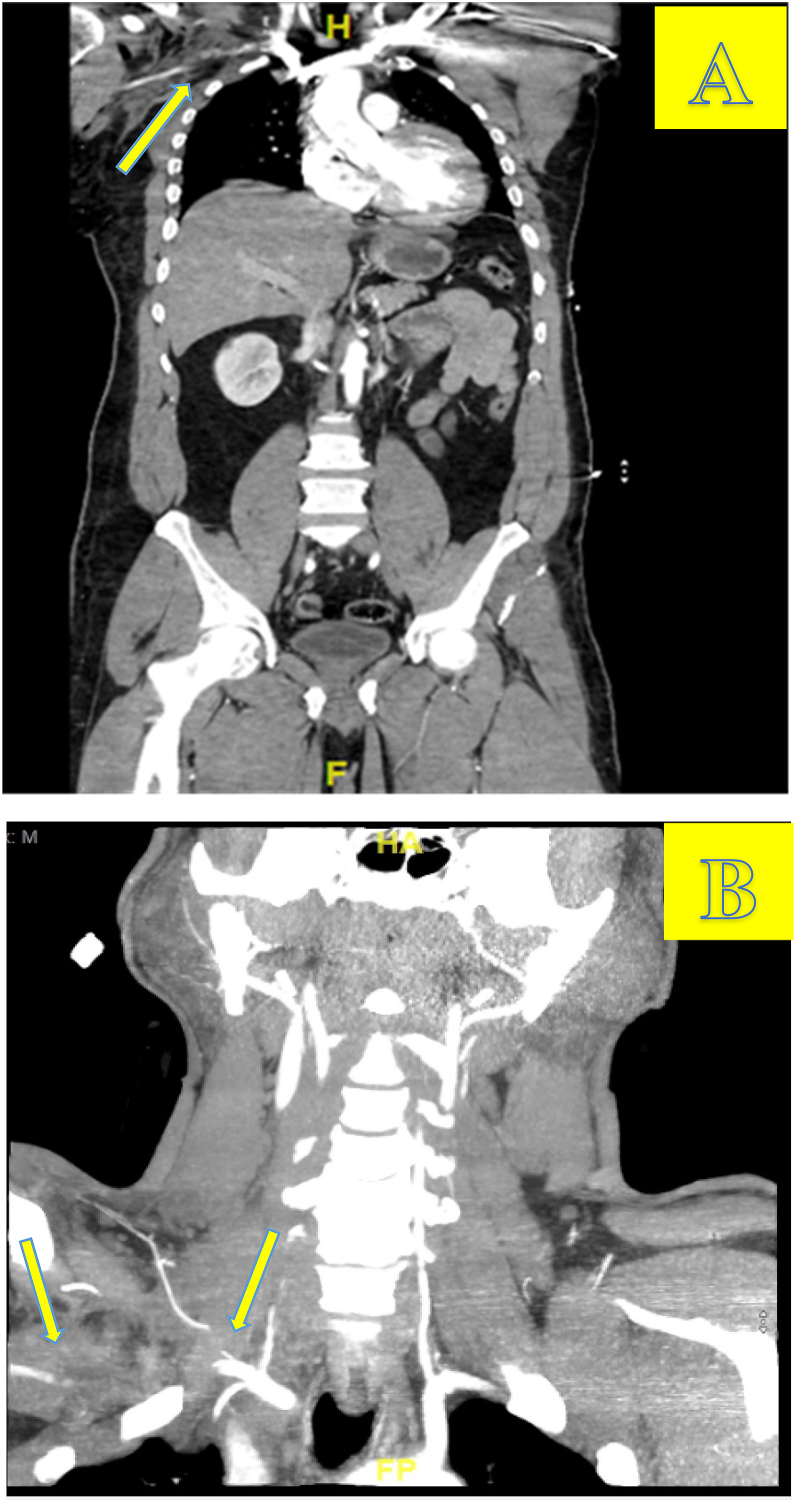
A: CT angiogram illustrating filling defect of the proximal right subclavian artery Fig. 3A legend. CT angiogram neck/R upper extremity showing traumatic occlusion of the Right subclavian a. just distal to the takeoff of the vertebral artery from a flap of intramural hematoma with a 6 cm flow gap distal to this injury. Distal reconstitution of the axillary artery just proximal to the superior thoracic origin is seen which remains patent through the brachial artery in the right upper arm. Hematoma, soft tissue swelling, and subcutaneous emphysema are also seen in this area due to the traumatic injuries. Fig. 3B ) CT angiogram neck/right upper extremity showing traumatic occlusion of the right subclavian artery Fig. 3B Legend: CT angiogram neck/Right upper extremity showing traumatic occlusion of the Right subclavian artery just distal to the takeoff of the vertebral artery; there is a 6 cm flow gap distal to this injury. Hematoma, soft tissue swelling, submit. emphysema are also seen in this area due to the traumatic injuries.

He was hemodynamically stable upon presentation and was able to undergo cross sectional imaging. CTA neck and right upper extremity showed an injury to the mid-right subclavian artery near the vertebral, thyrocervical, and internal thoracic arterial origins (Fig. 3A & B). There was traumatic occlusion of the subclavian artery beyond that point with a 6 cm flow gap. However, there was reconstitution of the axillary artery distal to the occlusion just proximal to the superior thoracic origin, which remained patent through the brachial artery in the right upper arm. The patient was also found to have multiple displaced fractures of the left hand, a right sternoclavicular dislocation, non-displaced right distal clavicular fracture, acromial clavicular joint ligamentous injury, segmental fractures of the mid and distal radial shaft, left anterior iliac wing displaced fracture, mildly displaced fractures of the C2, 3, and 7 transverse processes and significant pulmonary contusions, all further illustrating the severity of his traumatic accident.

**Fig. 4:**
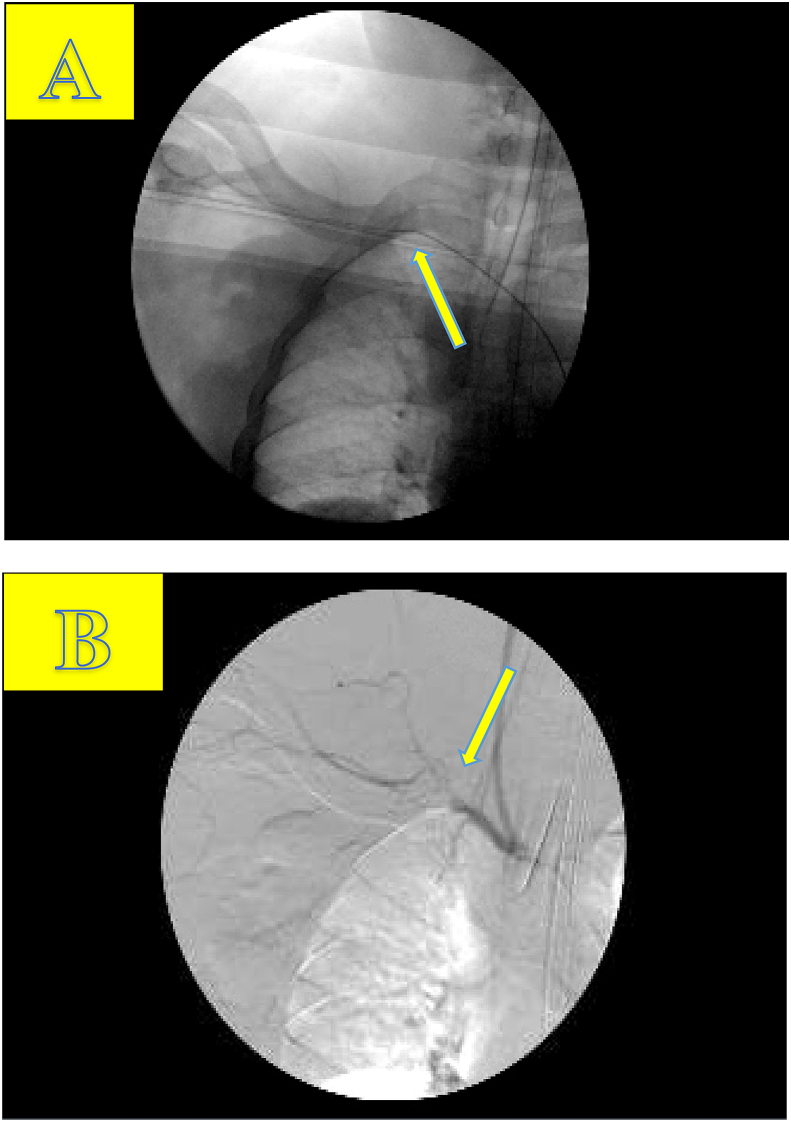
A. Angiogram R subclavian artery with wire seen into the R subclavian artery. Attempt was made bridge the defect/gap/tear of the subclavian artery, which was unsuccessful, and therefore open approach was then pursued. Fig. 4 B Angiogram intra-op done by fluoroscopy showing the R subclavian artery with injury to the subclavian artery; disruption of flow is seen on the angiogram.

**Fig. 5:**
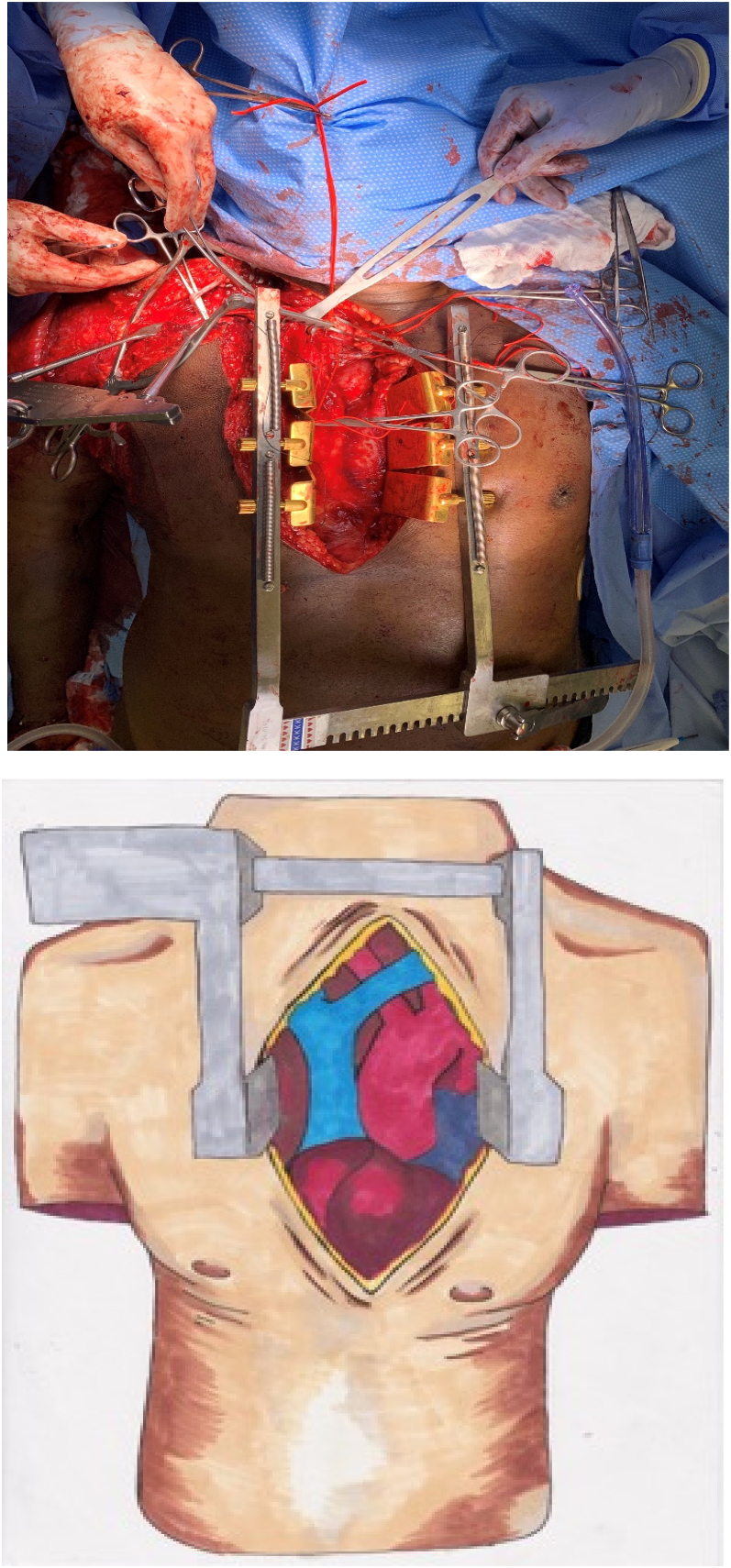
A: Median sternotomy with supraclavicular transverse incision including transection of the sternocleidomastoid and anterior scalene muscles Figure 5B: Diagram of median sternotomy approach.

After the completion of initial imaging, the patient was emergently taken to the operating room in an attempt to repair the right subclavian vasculature. A preliminary angiogram performed in the operating room revealed the defect suggested by CTA. Subsequent attempts to pass a guide wire through the subclavian occlusion proved futile (Fig. 4A & B). Based on the proximal position of the right subclavian defect, a surgical approach consisting of median sternotomy with supraclavicular transverse incision was performed for adequate right subclavian artery exposure (Fig. 5A). Initial sternotomy localized the innominate artery and proximal subclavian artery. The proximal subclavian evaluation revealed a transected and thrombosed subclavian artery distal to the takeoff of the vertebral and mammary arteries and thyrocervical trunk. Three brachial plexus cords were also found to be injured along with a fractured 1st rib. The proximal and distal ends of the transected subclavian vessel were controlled to ensure hemostasis and subsequently debrided to eliminate any remaining damaged tissue. After debridement, an approximately 6 cm space remained creating unsuitable tension parameters for primary repair. Instead, a 6mm-diameter ringed PTFE graft was placed to adequately repair the defect. Perfusion of the vessel was confirmed by intra-operative doppler distal to the site of repair and over the brachial and radial arteries. A distal palpable pulse was also noted. Due to the severity of his right upper extremity injury, a fasciotomy was performed at that time. Overall, his estimated blood loss was approximately 1.5 L.

The remainder of his injuries were managed with various surgeries performed by the orthopedics, trauma, and plastic teams. His post-operative course was associated with a significant ICU stay and acute respiratory failure secondary to pulmonary contusions. This resulted in a period of ventilator dependence with eventual requirement of a temporary tracheostomy. Overall, the patient had a total of 10 surgeries throughout his hospital course. He stayed in the ICU for approximately one month prior to being weaned off ventilator support. He was then transferred to the floor where he stayed for an additional month prior to discharge to a rehabilitation facility. On follow up in the trauma clinic, the patient was ambulating well and working with PT/OT at his rehabilitation facility. His tracheostomy was removed and he was able to speak in full sentences and tolerate an oral diet. The remainder of his clinical recovery was unremarkable.

## 3 Discussion

We present the case of a 50-year-old male with transection of the right subclavian artery secondary to severe blunt trauma who was successfully treated with bypass graft repair via median sternotomy approach. There are few documented cases regarding subclavian artery injuries secondary to blunt trauma. In those that do discuss injury to subclavian vessels, statistics show that blunt causes are significantly outnumbered by those secondary to penetrating mechanisms. A case series conducted by Costa et al. reported that out of 167 patients treated for confirmed subclavian/superior mediastinal artery injury, only 15 patients (9%) had such injuries due to blunt trauma. Of these 15, ten had injury to the proximal areas of the subclavian vessel. Hoff et al. also reported that over a ten-year period, 21 patients presented with innominate or subclavian artery injury with only 8 being secondary to blunt trauma. More commonly, subclavian injury and pseudoaneurysm formation is due to iatrogenic manipulation (central venous catheters, PCI, etc.) [4]. The underrepresentation of blunt subclavian trauma may be due in part to the severity of trauma required to have such injury and possible early field death. Enamorado et al. proposed that pre-hospital mortality rate of those with blunt subclavian injury may be as high as 60% at the event site, with a subsequent estimate of 5–30% in-hospital mortality due to hemorrhage or concomitant traumatic brain injury. These high mortality rates prior to adequate workup may impact the true statistics of the prevalence of these injuries. Overall, they estimate that blunt and penetrating subclavian injuries represent a mere 5% of all traumatic vascular lesions with a significant minority of these being secondary to blunt trauma.

Costa et al. showed that in all cases of blunt subclavian injury, concomitant injury to the thorax was found. The most common associated injury was rib fracture (11/15), with four of these involving the first rib as seen in our patient. Additionally, total brachial plexus disruption was seen in 9 of the 15 patients with blunt injury. This associated injury was seen to a lesser degree in our patient (only three cords were injured in our patient). Of note, all cases with injury to the distal subclavian artery had associated clavicular fracture. Our patient also had a non-displaced clavicular fracture and sternoclavicular dislocation. It is unclear whether these associated injuries are causative of, or merely correlated with, injury to the nearby subclavian vessels. The possibility of an independent mechanism of injury to the subclavian vasculature has been illustrated in cases of blunt subclavian injury without associated injuries. Enamorado et al. report a case of left subclavian artery pseudoaneurysm formation at its origin from the aortic arch secondary to blunt trauma experienced in a severe motor vehicle accident. Their rational for the isolated vascular injury was similar to that as previously documented by Binet et al. in which compressive forces of the chest wall in conjunction with rotational stress on the vasculature of the neck allow for the sheer force needed to sever the proximal mediastinal arteries from their origins. This idea of “sheer forces” leading to transection of arteries can be commonly seen in settings of vascular trauma secondary to joint dislocation or joint space disruption. For example, Elkbuli et al. recently report a case of an 80-year-old female who suffered axillo-subclavian dissection with pseudoaneurysm formation secondary to glenohumeral dislocation acquired via a simple ground level fall [7]. The sheer forces to the surrounding joint vasculature experienced in this setting may be similar to those experienced in proximal subclavian injury, albeit, in more severe trauma settings.

In general, appropriate management of subclavian injury is dependent on the mechanism and extent of injury. Often, these patients present with massive hemorrhage and require extensive resuscitation with rapid and emergent surgical management. Elkbuli et al. report use of endovascular balloon occlusion of a subclavian artery avulsion in order to achieve hemodynamic stability in a 30 year old male presenting with significant hemorrhage secondary to blunt trauma from a motorcycle accident. Our patient was hemodynamically stable on presentation and therefore was able to undergo cross sectional imaging prior to such interventions [9]. Due to the high pre-hospital mortality rate secondary to hemorrhage, our patient's presentation is rare in the context of severe subclavian injury with transection. Prior cases of penetrating subclavian injury have been reported in the literature and currently support a standard of open repair or graft placement to successfully manage penetrating vessel injury [5,10,11]. Such success of either approach likely depends on the severity/extent of the overall injury. In the setting of blunt trauma, Hoff et al. report a preference of graft placement over primary repair. As seen in our case, this may be due to the need for extensive debridement of transected and damaged arterial ends and subsequent excessive tension on an anastomosis if primary repair were attempted. New efforts to utilize an endovascular approach have also been reported to successfully manage both penetrating and blunt injuries to subclavian/axillary/brachiocephalic vasculature. Elkbuli et al. reported successful endovascular management in both penetrating and blunt injuries to the thyrocervical trunk, subclavian and axillo-subclavian arteries, respectively [7,8,12]. In all cases, control of pseudoaneurysm and extensive hemorrhage were adequately managed with embolization or endovascular stent placement. Despite emerging opportunities for successful minimally invasive endovascular approaches, many subclavian injuries require significant open intervention for adequate management. Interestingly, Posner et al. concluded that open anatomic repair is the most desirable surgical option to prevent possible future subclavian steal syndrome, claudication or ischemia resulting in limb amputation. Ligation alone has many of these complications despite collateral circulation being seemingly sufficient. In our presented case, our patient initially had a pulse deficit of the right upper extremity, which was confirmed by Doppler (implying inadequate collateral circulation). An attempt to pass the catheter wire past the subclavian defect was unsuccessful, further justifying the need for an open repair rather than attempting an endovascular ligation or stent placement.

As seen in our case, the necessity for open repair of blunt subclavian injury is often apparent. At that point, the remaining crucial decision is which surgical approach will best target the affected site with minimal time wasted making unnecessary/ineffective incisions in attempt to gain access. Such decisions require a solid understanding of the complex underlying anatomy and accurate localization of the arterial defect prior to operation. Our patient received immediate angiography upon arrival to the OR and was confirmed to have a complete transection of the proximal right subclavian artery near its origins from the innominate artery. Due to the proximal location and extent of the defect, a median sternotomy with extension to a right supraclavicular incision was utilized to best visualize the injury and obtain adequate control for successful graft repair. Extension of the incision to the supraclavicular space was imperative, as the median sternotomy alone was insufficient for controlling the distal aspect of the transected subclavian vessel. This approach differs from proximal left subclavian injuries in which anterolateral or posterolateral thoracotomy can allow adequate access to the target vasculature and its origins at the aorta. The difference of approach is due to the unique anatomic distribution of the right subclavian (a continuation of the innominate artery that roughly crosses the midline adjacent to the sternum) compared to that of the left subclavian (a direct branch of the aortic arch, whose view is often not hindered proximally by the sternum (Fig. 5B) [13]. Regardless, median sternotomy has been utilized in previous left proximal subclavian injuries as well but is not necessary in all cases [14]. Our approach mirrors that previously documented by Schaff and Brawley in 1977 in which median sternotomy was successfully utilized in the management of six patients with proximal subclavian/neck injuries acquired secondary to penetrating trauma. Additional cases of median sternotomy in successful management of penetrating right subclavian injuries have since been documented with rather low rates of perioperative complications, but most of these cases have had minimal follow up to identify the long-term effects of such interventions [14]. Kapetanakis et al. have also previously reported use of median sternotomy for successful graft repair of a partially transected proximal right subclavian artery injury acquired secondary to blunt trauma. To our knowledge, this is the only other documented case describing median sternotomy for surgical management of a blunt right subclavian injury. Overall, mortality rates of blunt right subclavian injury appear to be high. However, rapid intervention with open repair via median sternotomy and subsequent graft placement in those who survive to in-hospital evaluation appears to significantly reduce risk of mortality and provide opportunity for complete functional restoration in these patients. Our case report highlights an extremely rare phenomenon that when described in literature, often results in rapid patient demise before surgical intervention is feasible to attempt. Our patient's full recovery in the context of the prevailing literature suggests that the surgical approach utilized was essential in adequately preventing his otherwise inevitable morality. Successful intervention of severe proximal subclavian injuries with median sternotomy is not commonly observed. However, such intervention in those cases that do present have proven lifesaving.

## 4 Conclusion

Blunt injury to the proximal right subclavian artery is a rare phenomenon, and when present is typically associated with other major injuries. Prompt identification of arterial injury is paramount as such injuries carry high rates of mortality and morbidity. Median sternotomy with supraclavicular extension is an appropriate open surgical approach to successfully manage severe, proximal right subclavian artery injuries. In the case of our patient, this approach allowed for direct visualization of the arterial defect, quick hemostatic control, and adequate graft repair leading to his otherwise unlikely survival and eventual functional recovery from the remainder of his injuries.

## Ethical **approval**

This report was conducted in compliance with ethical standards. Informed written consent has been obtained and all identifying information is omitted.

## Sources of funding

None.

## Author contributions

AE, KK, DB, JW– Conception of study, acquisition of data, analysis and interpretation of data.

JW, DB – Management of case AE, KK, BD, DB, MM, JW –drafting of abstract, drafting of manuscript, critical revision of manuscript.

AE, KK, BD, DB, MM, JW – Approval of the final version for submission.

## Registration of research studies

This is a case report study.

## Guarantor

Dessy Boneva.

John Whitehead.

## Provenance and peer review

Not commissioned, external peer review.

## Informed consent

Informed written consent has been obtained and all identifying information is omitted.

## Declaration of competing interest

No conflicts of interests.
